# In Vitro Activity of 22 Antibiotics against *Achromobacter* Isolates from People with Cystic Fibrosis. Are There New Therapeutic Options?

**DOI:** 10.3390/microorganisms9122473

**Published:** 2021-11-30

**Authors:** Clémence Beauruelle, Claudie Lamoureux, Arsid Mashi, Sophie Ramel, Jean Le Bihan, Thomas Ropars, Anne Dirou, Anandadev Banerjee, Didier Tandé, Hervé Le Bars, Geneviève Héry-Arnaud

**Affiliations:** 1 University Brest, INSERM, EFS, UMR 1078, GGB, 29200 Brest, France; claudie.lamoureux@chu-brest.fr (C.L.); Genevieve.Hery-Arnaud@univ-brest.fr (G.H.-A.); 2 Department of Bacteriology, Virology, Hospital Hygiene, and Parasitology-Mycology, Brest University Hospital, 29200 Brest, France; arsid.mashi@gmail.com (A.M.); didier.tande@chu-brest.fr (D.T.); herve.lebars@chu-brest.fr (H.L.B.); 3 Centre de Ressources et de Compétences de la Mucoviscidose, Fondation Ildys, Presqu’île de Perharidy, 29680 Roscoff, France; sophie.ramel@ildys.org (S.R.); jean.lebihan@ildys.org (J.L.B.); thomas.ropars@ildys.org (T.R.); anne.dirou@ildys.org (A.D.); anandadev.banerjee@ildys.org (A.B.)

**Keywords:** *Achromobacter*, non-fermenting Gram-negative bacilli, cystic fibrosis, antibiotic susceptibility, resistance, multidrug resistance

## Abstract

Bacteria belonging to the genus *Achromobacter* are increasingly isolated from respiratory samples of people with cystic fibrosis (PWCF). The management of this multidrug-resistant genus is challenging and characterised by a lack of international recommendations, therapeutic guidelines and data concerning antibiotic susceptibility, especially concerning the newer antibiotics. The objective of this study was to describe the antibiotic susceptibility of *Achromobacter* isolates from PWCF, including susceptibility to new antibiotics. The minimum inhibitory concentrations (MICs) of 22 antibiotics were determined for a panel of 23 *Achromobacter* isolates from 19 respiratory samples of PWCF. Two microdilution MIC plates were used: EUMDROXF^®^ plate (Sensititre) and Micronaut-S Pseudomonas MIC^®^ plate (Merlin) and completed by a third method if necessary (E-test^®^ or UMIC^®^). Among usual antimicrobial agents, the most active was imipenem (70% susceptibility). Trimethoprim-sulfamethoxazole, piperacillin and tigecycline (65%, 56% and 52% susceptibility, respectively) were still useful for the treatment of *Achromobacter* infections. Among new therapeutic options, β-lactams combined with a β-lactamase-inhibitor did not bring benefits compared to β-lactam alone. On the other hand, cefiderocol appeared as a promising therapeutic alternative for managing *Achromobacter* infections in PWCF. This study provides the first results on the susceptibility of clinical *Achromobacter* isolates concerning new antibiotics. More microbiological and clinical data are required to establish the optimal treatment of *Achromobacter* infections.

## 1. Introduction

Bacteria belonging to the genus *Achromobacter* are increasingly isolated from respiratory samples of people with cystic fibrosis (PWCF). In France, their prevalence raised from 3.1% in 1999 to 6.9% in 2019 [1]. This opportunistic pathogen has been associated with acute pulmonary exacerbations, chronic infections and higher concentrations of TNF-α in sputum samples. *Achromobacter* infection occurs most commonly in CF patients with advanced lung disease and is associated with a poor clinical course [2,3,4,5,6,7]. *Achromobacter* species are well-armed for colonisation and persistence in the CF lung, as they harbour a large panel of adaptive strategies (biofilm formation, antibiotic resistance, hypermutation, secretion and quorum sensing systems) [8]. However, the current evidence is insufficient to attribute a major role in disease progression and large prospective studies assessing the pathogenicity of this emerging pathogen are warranted. Today, there is no international standard concerning the management of *Achromobacter* lung infection (when to treat and how to treat), including in PWCF. Moreover, the introduction of an adapted antibiotic therapy is often difficult because of the numerous natural and acquired resistances that characterise the *Achromobacter* genus [9]. Resistance, either innate or acquired, is conferred by two major mechanisms: extrusion of the antibiotics through efflux pumps and enzymatic degradation [8,9]. At least two efflux pumps confer intrinsic resistances: the *AxyABM* efflux pump, which is mainly involved in the extrusion of cephalosporins, and the *AxyXY-OprZ* efflux pump, which is mainly involved in the extrusion of aminoglycosides. *Achromobacter* produces a constitutive β-lactamase (OXA-114) with activity against penicillin G, ticarcillin, piperacillin and cephalotin even though ticarcillin and pipercacillin susceptibility is common among *Achromobacter* isolates. The lack of therapeutic guidelines was accompanied, until recently, by a lack of technical guidelines for performing antibiotic susceptibility tests, including specific breakpoints. Consequently, most of the published data are based on the CLSI (Clinical and Laboratory Standards Institute) breakpoints for the “other non-Enterobacterales” category, which is not adapted to characterise *Achromobacter* susceptibility. In 2020, EUCAST (European Committee on Antimicrobial Susceptibility Testing) proposed a genus-specific susceptibility testing method and consensually accepted susceptibility breakpoints for three antibiotics (i.e., piperacillin-tazobactam, meropenem and trimethoprim-sulfamethoxazole [SXT]) [10]. Another recent change concerns the availability of new antibiotics. Indeed, new antibiotics with anti-Gram negative activity (as cefiderocol, eravacycline or new β-lactam-β-lactamase inhibitor associations) are recently available and could be of interest in the treatment of *Achromobacter* infections. 

Today, few data exist to describe the susceptibility of *Achromobacter* isolates to antibiotics, especially to the recently marketed molecules. Given this observation, the objective of our study was to describe the antibiotic susceptibility of *Achromobacter* in PWCF, including concerning new molecules. Therefore, we determined the minimum inhibitory concentration (MICs) of 22 antibiotics for a panel of 23 *Achromobacter* clinical isolates from respiratory samples of PWCF.

## 2. Materials and Methods

### 2.1. Patients and Isolates Characteristics

Twenty-three isolates of *Achromobacter* cultured from sputum of 19 PWCF followed at the Western Brittany CF centre (Roscoff, France) during the year 2020 have been included in this study. Sputum samples were collected during follow-up consultations as part of routine patient monitoring. Demographic data (sex and age) and state of *Achromobacter* colonisation were recorded. Patients were considered chronically colonised according to the criteria defined by Pereira [11] (at least three positive cultures in one year obtained with a minimum interval of one month between them, for at least two years). 

Microbial analysis was performed at the clinical bacteriology unit of Brest University Hospital following French recommendations [12]. Isolates identified as *Achromobacter* by matrix-assisted laser desorption/ionisation time of flight mass spectrometry (MALDI-TOF MS biotyper MBT with the Bruker’s library version 9 (Bruker, Bremen, Germany) were stored at −80 °C (Microbank, Pro-Lab Diagnostics, Richmond Hill, ON, Canada), and grown secondarily on Columbia agar with 5% horse blood (bioMérieux, Marcy l’Etoile, France). Freshly cultured isolates were spotted in duplicates onto MALDI-TOF MS target plates. A score of ≥1.7 was considered as an accurate genus-level identification. As MALDI-TOF MS is not reliable at distinguishing *Achromobacter* at the species level, we did not consider the species identified by MALDI-TOF [13].

### 2.2. Antibiotic Susceptibility Testing

Susceptibility testing of the isolates was performed as recommended by EUCAST 2021 [14]. Briefly, an inoculum of 0.5 McFarland was prepared in physiological water from a fresh culture and was used for the preparation of all the tests. The bacterial suspension was transferred in a cation-adjusted Mueller-Hinton (MH) broth (Merlin Diagnostika, GmbH, Bornheim, Germany). MIC plates based on microdilution were inoculated according to the manufacturer’s recommendations: EUMDROXF^®^ plate (Sensititre, Thermo Fisher Scientific, Cleveland, OH, USA) and Micronaut-S Pseudomonas MIC^®^ plate (Merlin Diagnostika, GmbH, Bornheim, Germany). The MICs of 22 antibiotics active on non-fermenting Gram-negative bacilli, listed in Table 1, were thus determined using the broth microdilution method. Given the dilution range for SXT contained in the plates, this association was also tested by the agar diffusion method. An MH agar plate (BioMerieux, Marcy-l’Etoile, France) was inoculated, and a disk of SXT (Bio-Rad, Marne-la-coquette, France) was tested, according to the EUCAST standardised disk diffusion method [15]. 

The MICs were determined as the lowest antibiotic concentration that completely inhibited visible bacterial growth after 20 ± 4 h incubation at 35 ± 2 °C in an aerobic atmosphere. MIC_50_ and MIC_90_ were calculated for the 22 antimicrobial agents [16]. The MIC_50_ and the MIC_90_ represent the MIC value at which ≥50% and ≥90% of the isolates are inhibited, respectively. Inhibition zone diameter was determined as the point where no obvious growth was detected or at the outer zone edge in case of a fine haze in the inhibition zone, following EUCAST recommendations [14]. All results were read in duplicate, independently, by two different people. In the case of a discrepancy between the two operators, a third person read the results. *Pseudomonas aeruginosa* ATCC 27853 was used as a control for the two microdilution MIC plates.

Results were interpreted according to EUCAST 2021 breakpoints for *A. xylosoxidans* for piperacillin-tazobactam, meropenem and SXT, for *Pseudomonas* for colistin and according to non-species-related pharmacokinetic/pharmacodynamic (PK/PD) breakpoints for the other antibiotics except for eravacycline for which there are no breakpoints. For eravacycine, the breakpoint for Enterobacterales was used (listed in Table S1). 

Results were also interpreted according to CLSI 2021 breakpoints for other non-Enterobacterales and for *P. aeruginosa* in the absence of breakpoints (for ceftazidime-avibactam, ceftolozane-tazobactam, imipenem-relebactam, cefiderocol and colistin) (Table S1) [17]. 

### 2.3. Comparison of Different Susceptibility Testing Methods

We measured the agreement between the two methods calculating the Cohen’s kappa (κ) considering susceptibility criteria according to EUCAST breakpoints [18]. The values of κ were interpreted according to the method of Landis and Koch, as follows: 1.00 to 0.81, excellent; 0.80 to 0.61, good (substantial); 0.60 to 0.41, moderate; 0.40 to 0.21, fair; 0.20 to 0.00, negligible agreement [19].

A mismatch between plates was considered acceptable if it involved only one dilution gap and unacceptable if it involved two or more dilutions gaps. In the case of unacceptable mismatch, a third method was used (MIC microdilution by UMIC^®^ (Biocentric, Bandol, France) for colistin or E-test^®^ (bioMerieux, Marcy-l’Etoile, France) for other antimicrobial agents). 

## 3. Results

### 3.1. Patients and Isolates Characteristics

A total of 23 isolates of *Achromobacter* cultured from sputum of 19 PWCF have been included. Among the 19 PWCF, 11 were chronically colonised, 4 were sporadically colonised and for the other four patients, the number of samples was insufficient to qualify patient colonisation. The sex ratio M/W was 1.7, and the median age was 27 years (range 9–47).

For four of the 19 PWCF, two strains were isolated simultaneously and were included in the study. 

### 3.2. Antibiotic Susceptibility Testing

The susceptibility testing results are summarised in Table 1, as well as MIC_50_ and MIC_90_ for each antimicrobial agent. The full MIC distributions for these isolates are available in Table S2. 

#### 3.2.1. Percentage of Susceptibility According to EUCAST Breakpoints 

The 23 isolates included in the study displayed the innate resistance to aztreonam, aminoglycosides and fosfomycin expressed by the majority of *Achromobacter* species (Table 1). 

Among the 22 antibiotics tested, the most active were cefiderocol (91% susceptibility with both plates) and imipenem (70% and 91%, respectively, with Merlin and Sensititre plates). Meropenem was less active than imipenem (respectively 48 vs. 70% [Merlin] and 43 vs. 91% [Sensititre]). The association meropenem-vaborbactam was active for 87% of isolates. Piperacillin alone or associated with tazobactam was active in more than half of the isolates (56%), as well as SXT (65%) and tigecycline (52%). Colistin remained susceptible for about one third of isolates (30% [Sensititre] to 39% [Merlin]). Resistance to ceftazidime was observed for the majority of isolates (17% susceptibility). The addition of avibactam raised the susceptibility to 26%. Cefepime, ceftolozane–tazobactam and fluoroquinolones were inactive for the majority of isolates (≤10% susceptibility) (Table 1). 

The addition of a β-lactamase inhibitor to a β-lactam appeared to provide a significant improvement for ceftazidime and meropenem with a raised susceptibility with avibactam and vaborbactam, respectively (17 to 26% for ceftazidime ± avibactam and 43 to 87% for meropenem ± vaborbactam). Nonetheless, MIC_50_ and MIC_90_ observed for ceftazidime or meropenem alone were similar or slightly different and generally concerned one MIC dilution to those obtained for ceftazidime-avibactam or meropenem-vaborbactam (Table 1 and Table S2). Concerning other β-lactams (piperacillin and imipenem), MIC_50/90_, percentage of susceptibility and MIC for each isolate were similar for β-lactam alone and β-lactam-β-lactamase inhibitor association (piperacillin-tazobactam or imipenem-relebactam). We can also notice a breakpoint difference between β-lactam alone and β-lactam inhibitors for these antibiotics (Table S1), which can explain susceptibility differences. 

Among new antimicrobial agents, eravacycline presented relatively low MICs (MIC_50/90_ = 0.5/>0.5 mg/L), similar to those of tigecycline (Table 1, Table S2). Considering a similar breakpoint from eravacycline and tigecycline, susceptibility was higher for eravacycline (65% vs. 52%). Nonetheless, the MIC_50/90_ of tigecycline (0.5/1 mg/L) was similar to those of eravacycline, and the difference of percentage of susceptibility was due to a one-MIC dilution difference for the two isolates (Table S2).

#### 3.2.2. Percentage of Susceptibility According to CLSI Breakpoints 

Different breakpoints to characterise the isolate are observed between the European and US standards. The antibiotics concerned are piperacillin, piperacillin-tazobactam, aztreonam, cefepime, ceftazidime, imipenem, meropenem, ciprofloxacin, levofloxacin, gentamicin, amikacin, tobramycin and SXT (Table S1). For these 13 antibiotics, the MIC value corresponding to the “susceptible” breakpoint is systematically higher in CLSI than in EUCAST. These differences of breakpoints reflect differences concerning percentages of susceptibility. Indeed, the percentage of isolates susceptible to these 13 antibiotics were higher with CLSI than with EUCAST (Table 1). In particular, this concerned levofloxacin (which raised from 4% to 48% with CLSI breakpoint), cefepime (≤10% from 22% [Sensititre plate]), ceftazidime (17% from 30%), meropenem (48% from 70% [Merlin]), amikacin (0% to 22% [Merlin]) and piperacillin-tazobactam (57% from 65%).

For three antibiotics (meropenem-vaborbactam, tigecycline and eravacycline), no breakpoints were available according to CLSI.

### 3.3. Comparison of Different Susceptibility Testing Methods

Among the 22 antibiotics tested, 11 were evaluated by both Merlin and Sensititre plates. Piperacillin, ceftazidime, ciprofloxacin, levofloxacin, gentamicin and SXT were tested only with the Merlin plate, whereas cefiderocol, imipenem–relebactam, meropenem–vaborbactam, tigecycline and eravacycline were evaluated only with the Sensititre plate (Table 1).

We compared the MICs obtained for the 11 antibiotics present in both plates. The agreement between the two microplates varied depending on the antimicrobial agent. An excellent agreement was obtained for piperacillin-tazobactam, aztreonam, ceftolozane-tazobactam, fosfomycin, amikacin and tobramycin (κ = 1) and meropenem (κ = 0.91), a substantial agreement for ceftazidime-avibactam, cefepime and colistin (κ = 0.71, 0.65 and 0.62, respectively) and a fair agreement for imipenem (κ = 0.36). 

Considering the dilution gap, a perfect match was observed for four of the 11 antibiotics. For six antibiotics, differences in MICs were observed but only with one dilution difference between the two plates, making these discrepancies negligible. In contrast, for three antibiotics (imipenem, meropenem and colistin), these discrepancies involved at least two dilutions (Table 2). The MICs for imipenem were systematically lower in Sensititre plates (five isolates, 2 mg/L vs. >8 mg/L), while the MICs for meropenem (two isolates) and colistin (one isolate) were lower in Merlin plates (0.5 vs. 2 mg/L, 4 vs. 16 mg/L [meropenem] and ≤1 vs. >16 mg/L [colistin]) (Table S2).

The SXT was present exclusively in the Merlin plate but the minimum concentration tested in the plate (≤1/19 mg/L) was higher than the EUCAST breakpoint (≤0.125 mg/L). The susceptibility of the SXT was evaluated by disk diffusion as a zone diameter breakpoint is available. A congruence was observed for 21 of the 23 isolates between MIC and zone diameter. In one case, disk diffusion categorised SXT as resistant (25 mm [breakpoint ≤ 26 mm] whereas MIC was ≤1/19 mg/L, which did not allow for interpreting the result). In one case, a discrepancy was observed between the two methods (MIC = 4 mg/L [resistant], diameter = 28 mm [susceptible]).

## 4. Discussion

Very few data are currently available about the susceptibility of *Achromobacter*, particularly with regard to new molecules. We propose here the description of the susceptibility of 23 *Achromobacter* isolates from PWCF respiratory samples by the determination of MICs of 22 antibiotics active on non-fermenting Gram-negative bacilli. MICs are determined using the broth microdilution method and interpreted according to EUCAST and CLSI. 

Among the usual antibiotics, imipenem (70% susceptibility according to the three methods used), meropenem (43% [Sensititre], 48% [Merlin]), piperacillin-tazobactam (57%), SXT (65%) and tigecycline (52%) demonstrated the greater activities, according to EUCAST. In contrast, ceftazidime and fluoroquinolones had poor activities (17% and ≤10% susceptibility, respectively). Considering CLSI breakpoints, susceptibility raised for levofloxacin (4% to 48%), cefepime (≤10% to 22%), ceftazidime (17% to 30%), meropenem (48% to 70%), amikacin (0% to 22%) and piperacillin-tazobactam (57% to 65%). Indeed, a large difference was observed according to the reference used, due to differences in breakpoints between the European and US standards. These discrepancies highlight the importance of specific breakpoints to the bacterial genus/species. This lack of specific breakpoints, combined with differences in breakpoints between the European and US standards, makes it difficult to interpret the results. Indeed, although these EUCAST *Achromobacter* specific breakpoints are currently only covering the top of the iceberg with just three antibiotics, they provide new perspectives essential to guide antimicrobial therapy and improve patient outcomes. Nonetheless, as the publication of specific EUCAST breakpoints is recent, the vast majority of the published data is based on the CLSI breakpoints. Indeed, considering CLSI breakpoints, European studies on the antibiotic susceptibility of *Achromobacter* isolated from PWCF have shown similar results with a good susceptibility of piperacillin-tazobactam (85 to 89%), imipenem (79 to 88%) and meropenem (54 to 72%) [20,21,22]. Conversely, US studies showed a lower susceptibility (piperacillin-tazobactam (13 to 55%), imipenem (59% and meropenem (28 to 51%)) [23,24].

We noticed that imipenem was more active than meropenem (MIC _50/90_ = ≤1/>8 vs. 2/>16 mg/L) as previously described [21,23,25]. These data are in contradiction with the EUCAST, which provides breakpoints for meropenem and proposes that imipenem has intrinsically insufficient activity for the breakpoint to be determined (TECOFF [tentative epidemiological cut-off values] of 8 mg/L) [10]. This is also the case for colistin, which shows an interesting activity in our study (39% susceptibility [Merlin]), and could represent an alternative in the case of pan-drug resistance. This discordance reflects, in our opinion, the lack of data concerning this bacterial genus. 

Among the associations of the β-lactam-β-lactamase inhibitor, the addition of a β-lactamase inhibitor does not appear to provide significant improvement. MIC_50_ and MIC_90 were_ observed for β-lactam alone, as well as the susceptibility percentage were similar to those obtained for β-lactam-β-lactamase inhibitor, except for meropenem-vaborbactam and ceftazidime-avibactam. Nonetheless, MIC differences observed between β-lactam alone and β-lactam-β-lactamase inhibitor were slight and concerned generally one MIC dilution. As in our study, Caverly et al. reported no difference between ceftazidime alone and ceftazidime-avibactam and a poor activity of ceftolozane-tazobactam [23]. They showed a better activity of meropenem-vaborbactam than meropenem alone (86% vs. 72%, breakpoint ≤4 mg/L) [23]. Given the fact that acquired carbapenem resistance occurs mostly via efflux systems and possibly by metallo-β-lactamases (MBLs) in *Achromobacter* species and that vaborbactam has no activity on these two mechanisms, this point needs to be clarified. 

Among new therapeutic options, cefiderocol appears to be particularly interesting. Cefiderocol has the advantage of being active on non-fermenting Gram-negative bacilli and of being relatively stable to nearly all β-lactamase enzymes, including MBLs [26]. In our study, cefiderocol showed activity on all but two isolates (91% susceptibility, MIC_50/90_ = 0.25/1 mg/L). In vitro data on cefiderocol activity against *Achromobacter* are very limited. Rolston et al. defined a MIC_90_ = 0.125 mg/L against 15 isolates from cancer patients [27]. This difference in MICs can be explained by at least two factors. The first factor identified is the characteristics of the patients in these studies. Our study concerns PWCF who receive numerous antibiotic therapies, and therefore, *Achromobacter* isolates are subject to high selective pressure. Secondly, we used a standard cation-adjusted MH broth, following the manufacturer recommendation for the inoculation of the Sensititre plate and not an iron-depleted MH broth as suggested by EUCAST and CLSI. Cefiderocol relies on active iron transport for entry into the periplasm, which is upregulated under iron-depleted conditions in vivo. Iron-depleted cation-adjusted MH broth mimics the in vivo condition. Initial studies demonstrated higher MICs when cefiderocol was tested with standard cation-adjusted MH broth than with iron-depleted broth [28]. Consequently, our MIC values may be overestimated. Concerning in vivo data, the use of cefiderocol in clinical case reports also suggest promising results [29,30].

Concerning eravacycline, a slight difference was noted between eravacycline and tigecycline but concerned only two isolates and one dilution gap. Eravacycline has demonstrated lower MIC than tigecycline for other non-fermenting Gram-negative bacilli as *Stenotrophomonas maltophilia,* but no *Achromobacter* isolate was included in the study [31]. Indeed, more studies are required to evaluate eravacycline activity on *Achromobacter.*

Currently, there are no official recommendations for the treatment of *Achromobacter* infections. The antibiotic therapy proposed is based on the combination of ciprofloxacin and carbapenem or on the combination of chloramphenicol and minocycline [32]. In our study, although imipenem demonstrated a good activity, all isolates were resistant to ciprofloxacin. Consequently, this association does not seem to be relevant. Both chloramphenicol and minocycline were not evaluated in our study. Due to its toxicity, chloramphenicol is rarely used. We tested tigecycline, a derivative of minocycline, which appears to demonstrate a good activity against *Achromobacter* (MIC_50_ = 0.5 mg/L and 1 mg/L for tigecycline and minocycline, respectively) [33,34]. Overall, clinical studies are needed to make recommendations for the treatment of *Achromobacter*, and more generally of non-fermenters, especially considering the increase in their prevalence and the recent availability of new antimicrobial agents. 

As *Achromobacter*, other non-fermenting Gram-negative bacilli, such as *Stenotrophomonas maltophilia*, *Burkholderia cepacia complex*, *Pandoraea* or *Cupriavidus* are increasingly isolated from the respiratory tract of PWCF and are characterised by their high resistance to antibiotics. For all these bacteria, SXT and tetracycline (minocycline) are among the most active antimicrobial agents [8,22,34,35,36,37,38]. For most of them, carbapenems represent one of the most active antimicrobial agents except for *S. maltophilia,* which is intrinsically resistant due to a chromosomal carbapenemase. Among newer antimicrobial agents, new β-lactam–β-lactamase inhibitor associations do not seem to cause any activity for *S. maltophila* or *Pandoraea,* as we described for *Achromobacter* spp. [23] (Table 3). However, β-lactam–β-lactamase inhibitor seems to be relevant for *Burkholderia* spp treatment [23]. Cefiderocol exhibits a great activity also against other non-fermenters [39,40]. Although clinical studies are needed to confirm the relevance of cefiderocol in the management of non-fermenters infections in CF, this antimicrobial agent seems to represent an interesting alternative to older agents.

In this study, we used two MIC plates in microdilution (Sensititre and Merlin). These two plates were complementary in terms of composition. Some molecules were redundant and allowed us to compare the results of the two plates. In the majority of cases, a perfect match was observed between both plates. Eight mismatches were observed between Merlin and Sensititre plates and concerned carbapenems (*n* = 7, κ = 0.36 for imipenem) and colistin (*n* = 1, κ = 0.62). This can probably be explained, at least in part, by the reading difficulties we encountered. The reading of MICs was sometimes difficult due to the non-homogeneous appearance of the well, particularly for colistin (Figure S1). In order to limit this bias, the data were systematically read in duplicate. Moreover, colistin has recently been the subject of a warning from EUCAST due to the difficulties of performing MIC determinations. However, the two plates we used were approved by EUCAST and showed good results compared to the reference technique [42]. These reading difficulties could also be responsible for the discrepancies between MIC and the inhibition zone diameters for SXT. Indeed, the fine haze in the inhibition zone made the reading of the diameter around the SXT sometimes difficult. 

Several limitations of this study should be acknowledged. First, we tested a limited number of isolates, all from the same CF centre. This study should be confirmed on a larger number of isolates coming from different geographical locations. Second, we did not perform molecular methods to explore the mechanism of resistance or to identify the isolates at the species level. Finally, although we used two MIC plates simultaneously, some molecules were only present in one of the plates, and we did not duplicate the tests.

## 5. Conclusions

The existence of numerous natural and acquired resistances that characterise the *Achromobacter* genus is associated either with the lack of specific breakpoints or with differences in breakpoints between the European and US standards, making it difficult to interpret the susceptibility results. The recent publication of specific breakpoints by EUCAST provides new perspectives essential to guiding antimicrobial therapy and guidelines for the management of *Achromobacter* infections. Among “older” antimicrobial agents, carbapenems, especially imipenem, piperacillin, SXT and tigecycline are still useful antibiotics for the treatment of *Achromobacter* infections. Among new therapeutic options, β-lactams combined with a β-lactamase inhibitor does not have benefits. On the other hand, cefiderocol appears as a promising therapeutic alternative for managing *Achromobacter* infections in PWCF. More data, including clinical data, are now required to establish the optimal treatment of *Achromobacter* infections.

## Figures and Tables

**Table 1:** In vitro activities of antimicrobial agents against Achromobacter isolates.

	MIC (mg/L)		Percentage of Susceptibility According to the Following Breakpoints			
	50%/90%		EUCAST ^c^		CLSI ^d^	
Antimicrobial agent	Merlin ^a^	Sensititre ^b^	Merlin ^a^	Sensititre ^b^	Merlin ^a^	Sensititre ^b^
Piperacillin	≤4/>32	-	57	-	61	-
Piperacillin—tazobactam	4/>128	≤4/>32	57	57	65	65
Aztreonam	>16/>16	>32/>32	0	0	0	0
Cefepime	>8/>8	>16/>16	4	9	13	22
Ceftazidime	>32/>32	-	17	-	30	-
Ceftazidime—avibactam	>8/>8	16/>16	26	39	26	39
Ceftolozane—tazobactam	>8/>8	>8/>8	0	9	0	9
Cefiderocol	-	0.25/1	-	91	-	91
Imipenem	≤1/>8	≤1/2	70	91	70	91
Imipenem—relebactam	-	1/2	-	91	-	91
Meropenem	2/>16	2/>16	48	43	70	57
Meropenem—vaborbactam	-	1/16	-	87	-	NA
Ciprofloxacin	4/8	-	0	-	4	-
Levofloxacin	4/8	-	4	-	48	-
Colistin	8/>8	16/>16	39	30	39	30
Fosfomycin	>128/>128	>64/>64	0	0	NA	NA
Gentamicin	>32/>32	-	0	-	9	-
Amikacin	>32/>32	>32/>32	0	0	22	13
Tobramycin	32/>32	>4/>4	0	0	9	13
SXT ^e^	≤1/>8	-	65	-	65	-
Tigecycline	-	≤0.5/1	-	52	-	NA
Eravacycline	-	0.5/>0.5	-	65	-	NA

The MICs were determined using two MIC plates in microdilution: ^a^ EUMDROXF^®^ plate (Sensititre) and, ^b^ Micronaut-S Pseudomonas MIC^®^ plate (Merlin). ^c^ MIC were interpreted according to EUCAST breakpoints defined for *A. xylosoxidans* for piperacillin-tazobactam, meropenem and trimethoprim-sulfamethoxazole (SXT), for *Pseudomonas* for colistin, for Enterobacterales for eravacycin and according to pharmacokinetic/pharmacodynamic (non-species-related) EUCAST breakpoints for the other antibiotics. ^d^ Results were interpreted according to CLSI breakpoint defined for other non Enterobacterales and for *P. aeruginosa* in the absence of breakpoints (for ceftazidime-avibactam, ceftolozane-tazobactam, imipenem-relebactam, cefiderocol and colistin). ^e^ SXT interpretation was based on the disk diffusion method and MIC values. NA: not applicable (absence of breakpoints).

**Table 2:** Concordance between the two microdilution plates (Merlin and Sensititre).

Antimicrobial Agent	κ	Perfect Match	1 Dilution	2 Dilutions	>2 Dilutions
Piperacillin—tazobactam	1	23			
Aztreonam	1	23			
Cefepime	0.65	20	3		
Ceftazidime—avibactam	0.71	20	3		
Ceftolozane—tazobactam	1	21	2		
Imipenem	0.36	19		5	
Meropenem	0.91	20	1	2	
Colistin	0.62	19	3		1
Fosfomycin	1	23			
Amikacin	1	22	1		
Tobramycin	1	23			

We measured the agreement between the two methods calculating the Cohen’s kappa (κ), considering susceptibility criteria according to EUCAST breakpoints. We also considered the number of dilution gaps for MIC. For the eight discrepancies (≥2 dilutions gap), we performed a third method (E-test^®^ for imipenem and meropenem and UMIC^®^ for colistin). The MICs obtained were between the values of the two plates for imipenem (6 mg/L for four isolates and 4 mg/L for one isolate). For meropenem and colistin, the MICs obtained by the third method were lower than those obtained by Merlin and Sensititre plates (0.125 and 2 mg/L [meropenem], 0.5 mg/L [colistin]) (Table S2).

**Table 3:** Activity of newer antimicrobial agents and comparator against non-fermenting Gram-negative bacilli.

Bacteria	Antimicrobial Agent (% Susceptibility)						
	Ceftazidime	Ceftazidime- Avibactam	Ceftolozane- Tazobactam	Meropenem	Meropenem- Vaborbactam **	Cefiderocol	References
*Achromobacter* spp.	30–71%	26–78%	1–10%	43–72%	65–86%	91–97%	[23,36,40,41]; Present study
*Burkholderia* spp.	20–91% *	24–97%*	12–89% *	90–100%	97–100%	75–94%	[23,36,40,41]
*S. malotphilia*	34%	27–48%	25–27%	0–11%	0–12%	100%	[23,36,40,41]
*Pandorae* spp.	0%	0%	0%	0%	0%	-	[23,36,40,41]
*Cupriavidus* spp.	23–27%	69–73%	73–90%	8–18%	-	-	[35,36]

As great differences exist between the studies (non-species-specific breakpoints, differences between CLSI and EUCAST breakpoints), percentage of susceptibilities are difficult to compare. * Lower susceptibility for *B. gladioli*, ** breakpoint ≤ 4 µg/mL.

## Data Availability

Not applicable.

## Supplementary Materials

The following are available online at https://www.mdpi.com/article/10.3390/microorganisms9122473/s1, Table S1. The EUCAST and CLSI breakpoints used to interpret *Achromobacter* MICs in the study; Table S2. MICs values obtained for each antibiotic and for each isolate of this study; Figure S1. Picture of MIC plate result (A) with panel configuration (B) (Merlin) and picture of the inhibition zone diameter for SXT (C).
