# Supplementary material for: In Vitro Activity of 22 Antibiotics against Achromobacter Isolates from People with Cystic Fibrosis. Are There New Therapeutic Options?
Source: Microorganisms. 2021 Nov 30;9(12):2473. doi: 10.3390/microorganisms9122473 (PMC8703882; doi:10.3390/microorganisms9122473)
Supplement: Supplementary file 1 [file microorganisms-09-02473-s001.zip › 2021_Achromo microorga Table S1 review.pdf]

Table S1. EUCAST and CLSI breakpoints used to interpret *Achromobacter* MICs in the study.

Antimicrobial agent	Breakpoints EUCAST[a]	CLSI[b]
Piperacillin	≤8 / >16	≤16/≥128
Piperacillin -tazobactam	≤4 />4	≤16/≥128
Aztreonam	≤4/ >8	≤8/32
Cefepime	≤4/ >8	8/32
Ceftazidime	≤4/ >8	8/32
Ceftazidime - avibactam	≤8/ >8	≤8/≥16
Ceftolozane - tazobactam	≤4/ >4	≤4/≥16
Cefiderocol	≤2/ >2	≤4/≥16
Imipenem	≤2/ >2	≤4/16
Imipenem - relebactam	≤2/ >2	≤2/≥8
Meropenem	≤1 / > 4	≤4/16
Meropenem - vaborbactam	≤8/ >8	NA
Ciprofloxacine	≤0.25/ >0.5	≤1/≥4
Levofloxacine	≤0.5/ >1	≤2/≥8
Colistin	≤2/ >2	≤2/≥4
Fosfomycin	≤8/ >8	NA
Gentamicin	≤0.5/ >0.5	4/16
Amikacin	≤1/ >1	16/64
Tobramycin	≤0.5/ >0.5	4/16
SXT[c]	≤ 0.125 / > 0. 125	2/4
Tigecycline	≤0.5/ >0.5	NA
Eravacycline	0.5	NA

[a]MIC were interpreted according to EUCAST breakpoints defined for *A. xylosoxidans* for piperacillin-tazobactam, meropenem and trimethoprime-sulfamethoxazole (SXT), for *Pseudomonas* for colistin, for Enterobacterales for eravacycin and according to pharmacokinetic/pharmacodynamic (non-species-related) EUCAST breakpoints for the other antibiotics. [b] Results were interpreted according to CLSI breakpoint defined for other non-Enterobacterales and for *P. aeruginosa* in the absence of breakpoints (for ceftazidime-avibactam, ceftolozane-tazobactam, imipenem-relebactam, cefiderocol and colistin). [c]SXT interpretation was based on disk diffusion method and MIC values. Different CLSI *vs* EUCAST breakpoints for sensitivity have been highlighted in grey. NA: not applicable.

Table S2. MICs values obtained for each antibiotic and for each isolates of this study. The MICs were determined using two MIC plates in microdilution: [a]the Micronaut-S Pseudomonas MIC® plate (Merlin) and [b]EUMDROXF® plate (Sensititre). For trimethoprim-sulfamethoxazole (SXT), a disk diffusion method was also used and results are expressed in mm corresponding to the inhibition zone diameter. MICs were interpreted according to EUCAST breakpoints as defined in Table S1, using a color code (green: susceptible, red: resistant, orange: intermediate, grey: uninterpretable).
