# Supplementary material for: In Vitro Activity of 22 Antibiotics against Achromobacter Isolates from People with Cystic Fibrosis. Are There New Therapeutic Options?
Source: Microorganisms. 2021 Nov 30;9(12):2473. doi: 10.3390/microorganisms9122473 (PMC8703882; doi:10.3390/microorganisms9122473)
Supplement: Supplementary file 1 [file microorganisms-09-02473-s001.zip › 2021_microorganism supdata3 review.pdf]

Figure S1. Picture of MIC plate result (A) with panel configuration (B) (Merlin) and picture of the inhibition zone diameter for SXT (C).

A

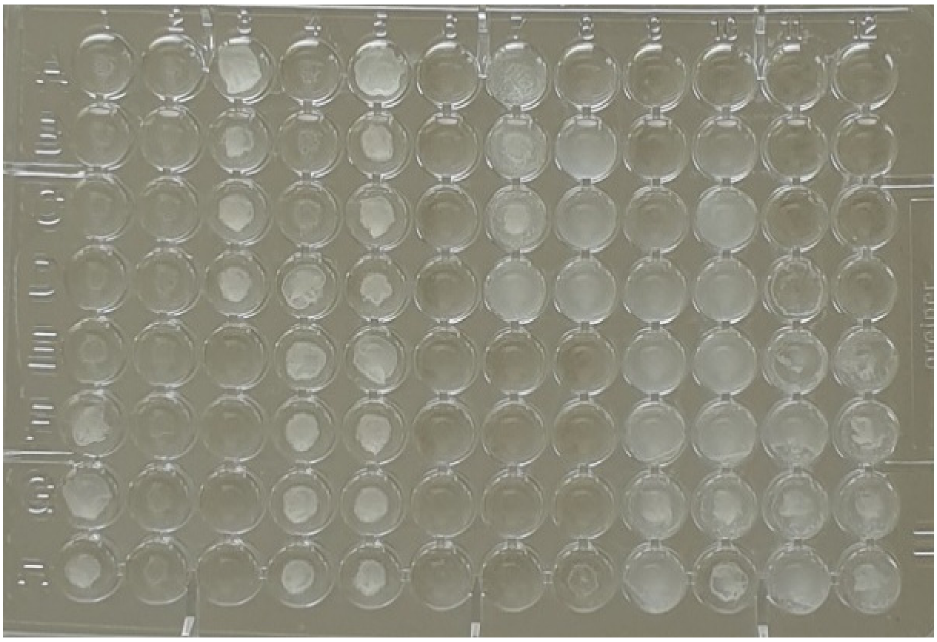

B

	1	2	3	4	5	6	7	8	9	10	11	12
A	PIP 32	PIT 128/4	AZT 16	CAZ 32	CEP 8	MER 16	FOS 128	AMK 32	TOB 32	GEN 32	CIP 8	LEV 8
B	PIP 16	PIT 64/4	AZT 8	CAZ 16	CEP 4	MER 8	FOS 64	AMK 16	TOB 16	GEN 16	CIP 4	LEV 4
C	PIP 8	PIT 32/4	AZT 4	CAZ 8	CEP 2	MER 4	FOS 32	AMK 8	TOB 8	GEN 8	CIP 2	LEV 2
D	PIP 4	PIT 16/4	AZT 1	CAZ 4	CEP 1	MER 2	FOS 16	AMK 4	TOB 4	GEN 4	CIP 1	LEV 1
E	CAA 8/4	PIT 8/4	T/S 8/152	CAZ 2	CTA 8/4	MER 1	IMP 8	COL 8	TOB 2	GEN 2	CIP 0.5	LEV 0.5
F	CAA 4/4	PIT 4/4	T/S 4/76	CAZ 1	CTA 4/4	MER 0.5	IMP 4	COL 4	TOB 1	GEN 1	CIP 0.25	LEV 0.25
G	CAA 2/4	PIT 2/4	T/S 2/38	CAZ 0.5	CTA 2/4	MER 0.25	IMP 2	COL 2	TOB 0.5	GEN 0.5	CIP 0.125	LEV 0.125
H	CAA 1/4	PIT 1/4	T/S 1/19	CAZ 0.25	CTA 1/4	MER 0.125	IMP 1	COL 1	TOB 0.25	GEN 0.25	CIP 0.0625	GC

C

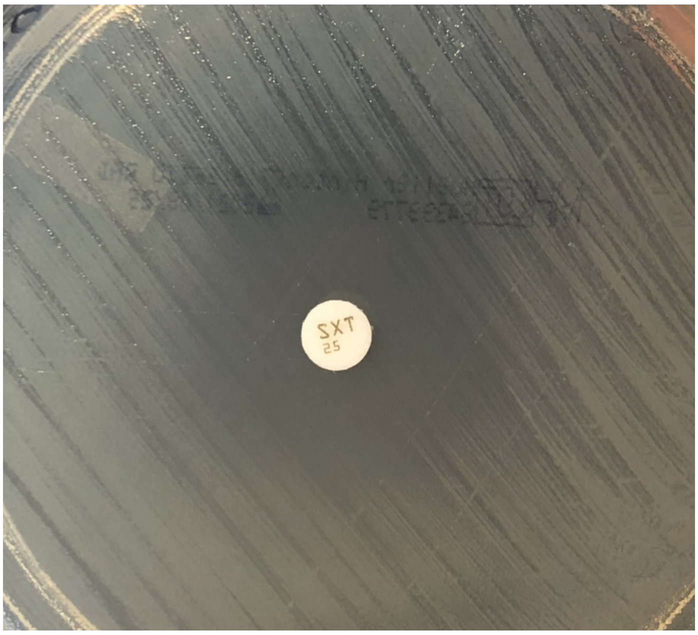

The reading of MICs was sometimes difficult, due to the non-homogeneous appearance of the well. Similarly, the fine haze in the inhibition zone made the reading of the diameter around the SXT sometimes difficult. PIP, piperacillin; CAA , ceftazidime-avibactam; PIT, piperacillin-tazobactam; AZT, aztreonam; T/S, SXT; CAZ, ceftazidime; CEP, cefepime; CTA, ceftolozane – tazobactam; MER, meropenem; FOS, fosfomycin; IMP, imipenem; AMK, amikacin; COL, colistin; TOB, tobramycin; GEN, gentamycin; CIP, ciprofloxacin, LEV, levofloxacin; GC, growth control
